# Association of Gene Polymorphism at Atrial Fibrillation in the Kazakh Population: Case—Control Study

**DOI:** 10.3390/genes17010084

**Published:** 2026-01-13

**Authors:** Dana Taizhanova, Nazira Bazarova, Akerke Kalimbetova, Roza Bodaubay, Elena Zholdybayeva, Chingis Abylkanov

**Affiliations:** 1 Department of Internal Diseases, Karaganda Medical University, 40 Gogolya Street, Karaganda 100000, Kazakhstan; taizhanova_kgma@mail.ru (D.T.); bodaubay.roza@gmail.com (R.B.); 2 Administration Department, Regional Clinical Hospital of the Karaganda Region Health Department, 41 Yerubayev Street, Karaganda 100000, Kazakhstan; bazarovazakirova@mail.ru; 3 Heart Rhythm Research Institute, Astana Medical University, 49A Beibitshilik Street, Astana 010000, Kazakhstan; 4 National Scientific Shared Laboratory of Biotechnology, National Center for Biotechnology, 13/5, Kurgalzhynskoye Road, Astana 010000, Kazakhstan; zholdybayeva@biocenter.kz; 5 Cardiology Department, Regional Clinical Hospital of the Karaganda Region Health Department, 41 Yerubayev Street, Karaganda 100000, Kazakhstan; abylkanov@gmail.com

**Keywords:** atrial fibrillation, gene polymorphism, Kazakh population, *PRRX1* gene, *rs3903239*, case—control study

## Abstract

Background/Objectives. Atrial fibrillation (AF) is the most common sustained cardiac arrhythmia and represents a major public health problem. Genetic factors contribute to AF susceptibility, including variants associated with atrial remodeling. Methods. This case–control study investigated the *rs3903239* polymorphism of the *PRRX1* gene in a Kazakh population. The main group included patients with AF (n = 75), the control group consisted of 2 subgroups: subgroup 1 (control group 1) included conditionally healthy patients (n = 73), subgroup 2 (control group 2) consisted of patients with arterial hypertension (AH) and coronary heart disease (CHD) without diagnosed AF at the time of inclusion in the study (n = 50). Genotype and allele frequencies were compared between patients with AF and two control groups. The frequency of the *rs3903239* polymorphism genotypes of the *PRRX1* gene in the main group and in the control groups was in the Hardy–Weinberg equilibrium. Results. The frequency of the rare G allele (AG + GG genotypes) was higher in patients with AF compared with conditionally healthy controls; however, this difference did not reach statistical significance (OR 1.357; 95% CI 0.845–2.178). Conclusions. The observed differences represent a non-significant trend and do not demonstrate a statistically confirmed association between the *rs3903239* polymorphism of the *PRRX1* gene and AF in the Kazakh population.

## 1. Introduction

Atrial fibrillation (AF) is the most common type of heart rhythm disorder, characterized by rapid and irregular excitation of the atrial myocardium. This arrhythmia leads to the disorganization of electrical activity, causing variability in the frequency of heart contractions and the duration of the cardiac cycle. Large-scale epidemiological studies in Europe and North America show that the prevalence of AF increases with age and is more often observed in men than in women. According to the latest studies (2020–2025), the global incidence of AF continues to grow, especially among the elderly, which is due to the improvement of diagnostic methods and the increase in life expectancy [1,2,3]. Recent data suggest that the prevalence of AF in the general population is approximately 2–4%, with incidence rising sharply to 10–17% in individuals aged 80 years and older [3,4]. Projections show that by 2050, the incidence of AF worldwide will more than double, posing significant challenges to health systems [4,5]. According to the results of a study in one of the regions of the Republic of Kazakhstan, the prevalence of AF among the population over 18 years of age was 3.3% [6,7].

The etiopathogenesis of AF is quite complex; studies conducted to date confirm the influence of both clinical factors on the development of AF and the genetic nature of the disease [8,9].

Currently, molecular studies of AF are focused on identifying genes and mutations that lead to arrhythmias (inheritance of such arrhythmias is carried out according to the classical Mendelian type), as well as on studying the polymorphism of various genes—the so-called candidate genes [10].

To date, 134 loci associated with AF have been identified. In 2012, a *GWAS* meta-analysis identified six new susceptibility loci, or about 10 likely candidate genes that exceeded the predetermined genome-wide significance threshold (*p* < 5 × 10^−8^) [6]. Based on the results of this meta-analysis, AF was found to have multiple genetic associations. The most significant association was found on chromosome *1q24* (*rs3903239*; overall *p* = 8.4 × 10^−14^) of the *PRRX* gene.

According to GeneCards.org, the *PRRX1* gene encodes a homeodomain transcription factor that is highly expressed in the developing heart, particularly in connective tissue. Biological interactions between the *PRRX1* gene and the related homeobox transcription factor gene *PRRX2* result in abnormal development of large vessels in a knockout mouse model [7]. These findings suggest that *PRRX1* gene expression may be involved in atrial structural remodeling processes that contribute to the development of atrial fibrillation.

The results of the studies depend on such factors as ethnicity, concomitant cardiac and extracardiac diseases, inheritance mechanisms, and intergenic interactions. The complexity of the etiopathogenesis and heterogeneity of AF pose the task of further searching for factors that play a leading role in the development of the disease. The currently available data on the association of candidate genes with AF are contradictory and necessitate more detailed studies in various ethnic groups. To date, gene polymorphism in AF has not been studied in populations living in Kazakhstan. It is not enough to simply project the data from foreign studies onto the large population of our country. Evaluation of the polymorphism of candidate genes associated with AF, depending on ethnicity, will open up new possibilities for a personalized approach to the prevention and therapy of patients with AF.

## 2. Materials and Methods

A total of 198 persons were included in the case–control molecular genetic study. The study was conducted at the clinical bases of Polyclinics No.3 RSE, Polyclinics No.5 RSE, Multidisciplinary Hospital No.1 RSE, and Multidisciplinary Hospital No 2 RSE in Karaganda. The main group included patients with AF (n = 75), the control group consisted of 2 subgroups: subgroup 1 (control group 1) included conditionally healthy patients (n = 73), subgroup 2 (control group 2) consisted of patients with arterial hypertension (AH) and coronary heart disease (CHD) without diagnosed AF at the time of inclusion in the study (n = 50). Inclusion criteria for the study: men and women of Kazakh nationality up to the third generation, long-term residents of the Republic of Kazakhstan; atrial fibrillation, documented by electrocardiography (ECG) or Holter 24-h ECG monitoring; signed informed consent; practically healthy; patients with hypertension and coronary heart disease without AF. Criteria for exclusion from the study: pregnant women; patients with chronic heart failure with functional class (FC) IV according to the NYHA classification; patients with acute cerebrovascular accident; patients with acute myocardial infarction; decompensated liver cirrhosis; severe renal dysfunction (SCF less than 15 mL/min); and patients’ refusal to participate in the study.

The scientific study was approved by the Ethics Committee of Karaganda Medical University (Protocol No. 14 dated 14 April 2020). All participants were included in the study after signing the informed consent form.

At the first stage, the analysis of anamnestic data, medical documentation, results of such clinical research methods as electrocardiography (ECG), Holter ECG monitoring, echocardiography (ECHOCG), selective coronary angiography (SCA), and laboratory research methods were carried out. In order to assess the risk factors for AF, a survey of patients included in the study was conducted. The questionnaire was developed based on the recommendations of the European Society of Cardiology (ESC) together with the European Association of Cardiothoracic Surgeons (EACTS) and included questions to identify clinical risk factors. The copyright for the development of the «Questionnaire for risk factors assessing atrial fibrillation» is attested in the State Register of Rights to Copyrighted Objects dated 16 November 2020, No. 13249.

A multivariable analysis adjusted for age was considered; however, due to the limited sample size, such an analysis could not be reliably performed. The study included patients with AF aged from 26 to 84 years. The average age of patients in the main group was 66.76 ± 9.13 (CI 64.66–68.86). A total of 57.3% of participants in the main group were male and 42.7% female. The largest group consisted of patients of the age group 61–70 years (n = 27) (36%), among whom 39.5% were male patients. Among females, the largest percentage (17%) were patients aged 71–80 years. The average age in the control group 1 was 54.32 ± 8.63, in the control group 2, 64.96 ± 9.74.

All patients with AF had episodes of this rhythm disorder lasting more than 30 s in the anamnesis or at the time of examination. Such episodes were recorded with ECG or Holter ECG monitoring.

To search for an association of gene polymorphism with AF in the Kazakh population, we selected the *rs3903239* polymorphism identified on chromosome *1q24* of the *PRRX* gene. The DNA-associated protein encoded by this gene is a member of the paired homeobox family localized in the nucleus. The protein acts as a transcriptional coactivator, enhancing the DNA-binding activity of serum response factor, a protein necessary for gene induction by growth and differentiation factors. The protein regulates creatine kinases in muscles, indicating its role in the formation of different types of mesodermal muscles.

DNA isolation was carried out in the Shared Laboratory of the Scientific Research Center of Karaganda Medical University. The DNA concentration was quantitatively measured using a spectrophotometric method on the NanoDrop1000 Thermoscientific spectrophotomet

The analysis of the *rs3903239* polymorphism of the *PRRX1* gene was carried out by a genotyping method based on polymerase chain reaction using a CFX 96 DNA amplifier (Bio-Rad, Hercules, CA, USA), detecting in real time. To prepare the samples, a set of primers was used—TaqMan^®^ SNP Genotyping Assay, Applied Biosystems, Waltham, MA, USA, and a commercial PCR mixture—Applied Biosystems^®^ TaqMan^®^ Universal PCR Master Mix. Genotyping was carried out at the shared-use laboratory of the National Center for Biotechnology in Astana.

The *rs3903239* polymorphism was identified as a result of a *GWAS* meta-analysis conducted in 2012 [6]. According to the results of this meta-analysis, this SNP showed a significant association on the *1q24* chromosome (*rs3903239*; total *p* = 8.4 × 10^−14^) of the *PRRX* gene.

The analysis of the results was carried out using the Bio-Rad CFX Manager 2 software (Bio-Rad Laboratories, Hercules, CA, USA). Based on the automatic detection of the accumulation level of PCR products by the device, accumulation curves were constructed along two corresponding channels of fluorophores. As a result, curves of different colors were built for one sample; the FAM fluorescence level curve was displayed in blue, and the VIC curve was displayed in red. For polymorphism in the *PRXX1* gene (*rs3903229* (A>G) homozygous genotype of the «wild» type), the VIC fluorescence level increased in the graph, but there was no increase in FAM. Conversely, if the FAM fluorescence level increased, but VIC did not increase, then this genotype was regarded as homozygous for the «mutant» type. If both fluorescence curves grew, then this genotype was heterozygous.

Statistical processing of the obtained data was carried out using the Statistica 6.0 software package. The assessment of the normality of the numerical variables’ distribution was realized using the Kolmogorov—Smirnov criteria. The data was analyzed at a significance level of α = 0.05. The description of quantitative data was carried out on the basis of the median and quartiles. For qualitative data, the proportion of persons with the characteristics of interest and a 95% confidence interval of the proportion determined by the Pearson χ^2^ method were calculated.

The frequency of the *rs3903239* polymorphism genotypes of the *PRRX1* gene in the main group and in the control groups was in the Hardy–Weinberg equilibrium. The frequency of the minor G allele occurrence in the Kazakh population corresponded to the frequency of the allele in the general population in accordance with the base 1000 genome G = 0.3393 (1699/5008, 1000 G).

The strength of the associations of the analyzed features was determined using the magnitude of the odds ratio. The odds ratio (OR) was considered statistically significant if the value was greater than or less than 1. If the confidence interval for the odds ratio included the number 1, then the calculated odds ratio would not be considered statistically significant.

The calculation of the odds ratio was used to identify the association between gene polymorphism and AF. The Pearson χ^2^ criterion was used to check the statistical significance of the differences between the «cases» and «controls» groups.

## 3. Results

The types of atrial fibrillation were determined according to the Clinical Guidelines for the Diagnosis and Treatment of Atrial Fibrillation, ESC 2020 [2].

Patients with permanent AF accounted for 63% (n = 47). Clinically, AF was manifested in paroxysmal form in 20% (n = 15) of cases, and it was persistent in 17% of cases (n = 13). The study included patients with an average score of 3.05 on the CHA2DS2-VAS scale; most of them had 2 points (n = 23) (30.7%). More than 3 points on the scale were noted in 40% (n = 30). The study included 50.7% (n = 38) patients with chronic heart failure (CHF) with FC ≤ I, 29.3% (n = 22) with FCII, 21.3% (n = 16) with CHF FC III. The decrease in the left ventricular ejection fraction (LVEF) of less than 40% was registered in 21.3% (n = 16) of patients, and LVEF ≥ 50% in 50.7% (n = 38). According to echocardiography, an increase in the left atrium (LA) of more than 50 mm in diameter was recorded in 56% (n = 42) of cases. In addition, the increase in the left ventricle of the heart was observed in 20% of cases, while in 16% (n = 12), normal dimensions of the heart chambers were observed on ultrasound examination of the heart.

In order to study the relationship of the *rs3903239* polymorphism of the *PRRX1* gene with the development of AF, 198 persons were examined, among whom 75 patients had AF. The control group consisted of 2 subgroups: subgroup 1 (control group 1) included conditionally healthy patients (n = 3), and subgroup 2 (control group 2) consisted of patients with arterial hypertension (AH) and coronary heart disease (CHD) without diagnosed AF at the time of inclusion in the study (n = 50). The initial characteristics of the study participants are presented in Table 1.

As Table 2 demonstrates, in the main group of patients with AF, the homozygous AA genotype for the common allele was detected in 33.3% of cases, the heterozygous AG genotype in 52% of cases, and the homozygous GG genotype for the rare allele in 14.7% of cases. In the group of conditionally healthy patients, the frequency of genotype occurrence was distributed as follows: the homozygous AA genotype—in 45.2%, the heterozygous AG genotype—in 42.5%, the homozygous GG genotype—in 12.3%. In the group of patients with AH and CHD, the frequency of genotype occurrence was as follows: the homozygous AA genotype—in 34%, the heterozygous AG genotype—in 48%, the homozygous GG genotype—in 18%. The frequency distribution of the minor rare G allele and the common A allele of the *rs3903239* polymorphism of the *PRRX1* gene in the main and the control groups was distributed as follows: the frequency of the rare G allele in the main group of patients with AF was 40.7%, and the frequency of the common A allele was 59.3%.

In the group of healthy patients, the frequency of the rare G allele was 33.6%, and the frequency of the common A allele was 66.4%. In the control group 2, the frequency of the rare G allele was 42%, and the frequency of the common A allele was 58% (Table 3).

Comparative analysis demonstrated a higher frequency of the rare G allele in patients with atrial fibrillation compared with conditionally healthy individuals. However, the calculated odds ratio did not reach statistical significance, as the 95% confidence interval included unity (OR 1.357; 95% CI 0.845–2.178). Therefore, the observed differences should be interpreted as a trend rather than evidence of a definitive genetic association (Table 4).

Thus, homozygous genotype AA according to the common allele was detected in 33.3% of cases, heterozygous genotype AG in 52% of cases, and homozygous genotype GG according to the rare allele in 14.7% of cases. In the group of conditionally healthy patients, the frequency of occurrence of genotypes was distributed as follows: homozygous genotype AA—45.2%, heterozygous genotype AG—42.5%, homozygous genotype GG—12.3%. The frequency distribution of the minor rare allele G and the common allele A polymorphism *rs3903239* of the *PRRX1* gene in the main and control groups was distributed as follows: the frequency of the rare allele G in the main group of patients with AF was 40.7%, and the frequency of the common allele A was 59.3%. In the group of healthy patients, the frequency of the rare allele G was 33.6%, and the frequency of the common allele AF was 66.4%. A comparative analysis of patients with AF with conditionally healthy patients showed that the homozygous genotype AA was less common in the group of patients with AF compared to control #1 (33.3% versus 45.2%, *p* < 0.05), and the homozygous genotype GG on the rare allele was more common in the group of patients with AF compared to the control group (14.7% versus 12.3%, *p* < 0.05). The frequency of the rare G allele in the main group of patients with AF was higher compared with healthy controls; however, this difference did not reach statistical significance (OR 1.357; 95% CI 0.845–2.178). Genotype distributions of the *rs3903239* polymorphism conformed to Hardy–Weinberg equilibrium in all study groups (AF group: *p* > 0.05; control group 1: *p* > 0.05; control group 2: *p* > 0.05).

## 4. Discussion

Screening for genetic variants and searching for genes associated with AF have been ongoing for many years. Linkage analysis and candidate gene sequencing have identified multiple polymorphisms in both familial and sporadic cases of AF [11,12,13,14]. As a result of studying the relationship between the polymorphism of the *RAAS* gene and the development of AF, Topal N.P. and co-authors (2011) genotyped the following polymorphisms: the I/D polymorphism of the *ACE* gene and the *M235T*, A-20C, and G-6A polymorphisms of the *AGTR* gene [15]. Ma R et al., in their meta-analysis, investigated the association between the I/D polymorphism of the *ACE* gene in association with AF. They observed the association between ethnicity and the I/D polymorphism of the *ACE* gene and concluded that individuals with the DD genotype of the *ACE* gene were at higher risk of developing AF [16]. The *ACE* gene *rs4343* (2350 G/A) polymorphism was analyzed in Chinese patients with hypertension. It was found that the 2350 G/A polymorphism was associated with AF, and the A allele determined an increased risk of developing AF in this group of patients [17]. In addition, Wang H et al. [18] found through meta-analysis that the *M235T* polymorphism of the *AGT* gene may be associated with an increased risk of developing AF in the Asian population. Most previous studies have focused on specific candidate genes associated with AF, while investigating the genetic basis of AF in the general population remains a more challenging task. To date, a significant advance has been the testing of common genetic variants that are more prevalent in populations of patients with AF compared to healthy controls in a number of case–control design association studies [15,16,19]. Significant progress in understanding the genetic basis of AF has been achieved with the advent of genome-wide association studies (*GWASs*). *GWASs* are large-scale genome-wide studies that involve genotyping of up to a million common variants or single-nucleotide polymorphisms (SNPs) and comparing their frequencies in patients with AF and controls [20]. These studies can identify genes that have not previously been implicated in AF, unlike case–control studies. To date, more than four large *GWASs* have been conducted in cohorts of patients with AF [21,22,23]. A total of 12 such studies had been conducted by 2018. In addition, a systematic review identified nine non-coding SNPs associated with an increased risk of AF. Large-scale genotyping in Europeans and Japanese identified new AF risk loci in or near the *NEURL, TBX5, CAND2, GJA1*, and *CUX2* genes [24,25].

According to the results of the largest *GWAS* meta-analysis conducted in 2012, the *rs3903239* polymorphism of the *PRRX1* gene was the most significant association on the 1q24 chromosome of the *PRRX* gene [8]. In 2018, the largest multiethnic study of the association of the genome with the left atrium in more than 500,000 participants, which included 84.2% European, 12.5% Japanese, 2% African American, and 1.3% Brazilian and Hispanic populations, confirmed the role of the *PRRX1* gene in the occurrence of AF [9]. In this regard, the purpose of our study was to identify the association of the *rs3903239* polymorphism of the *PRRX1* gene with the development of AF in the Kazakh population. We conducted a genetic study of 198 patients of Kazakh nationality, of whom 75 patients with AF were included in the main group. For comparison, the control group was formed of 73 conditionally healthy individuals who do not have diseases of the cardiovascular system and 50 patients with clinical and anamnestic data of AH and CHD disease without AF.

In the present study, we observed a higher frequency of the rare G allele and GG genotype of the *rs3903239* polymorphism of the *PRRX1* gene in patients with atrial fibrillation compared with conditionally healthy controls. However, these differences did not reach statistical significance, and the confidence intervals of the odds ratios included unity. Therefore, the results should be interpreted as indicating a non-significant trend rather than a confirmed genetic association.

The distribution of genotypes and alleles of the *rs3903239* polymorphism of the *PRRX1* gene was analyzed, and the obtained results were compared with data from patients belonging to the group of conditionally healthy individuals without cardiovascular pathology (control group 1). According to the results, in patients with AF, the homozygous AA genotype for the common allele was detected in 33.3% of cases, the heterozygous AG genotype in 52% of cases, and the homozygous GG genotype for the rare allele in 14.7% of cases. In the group of conditionally healthy patients, the frequency of genotype occurrence was distributed as follows: the homozygous AA genotype—in 45.2%, the heterozygous AG genotype—in 42.5%, the homozygous GG genotype—in 12.3%. The frequency distribution of the minor rare G allele and the common A allele of the *rs3903239* polymorphism of the *PRRX1* gene in the main and the control groups was distributed as follows: the frequency of the rare G allele in the main group of patients with AF was 40.7%, and the frequency of the common A allele was 59.3%. In the group of healthy patients, the frequency of the rare G allele was 33.6%, and the frequency of the common A allele was 66.4%. Comparative analysis of data from patients with AF with data from conditionally healthy patients showed that the homozygous AA genotype was less common in the group of patients with AF compared with the control group 1 (33.3% versus 45.2%, *p* < 0.05). The homozygous GG genotype for the rare allele in the group of patients with AF was more common compared with the control group (14.7% versus 12.3%, *p* < 0.05). The frequency of occurrence of the rare G allele in the main group of patients with AF prevailed in comparison with the group of healthy patients (40.7% versus 33.6%, *p* < 0.05, OR 1.357; 95% CI 0.845–2.178). The limitation of our study may be the small sample of patients with AF, so the obtained results, taking into account the CI/OR, are statistically insignificant.

An important limitation of this study is the relatively small number of patients with atrial fibrillation, which may have limited the statistical power to detect modest genetic effects. Consequently, the possibility of a type II error cannot be excluded. For this reason, the present study should be regarded as exploratory and hypothesis-generating, and the findings require confirmation in larger, well-powered cohorts.

Another important limitation of the study is the significant age difference between the atrial fibrillation group and the conditionally healthy control group. Given that age is a strong independent risk factor for atrial fibrillation, the lack of age-adjusted multivariable analysis due to the limited sample size may have influenced the observed associations and should be taken into account when interpreting the results.

## 5. Conclusions

In this case–control study conducted in a Kazakh population, the *rs3903239* polymorphism of the *PRRX1* gene showed a non-significant trend toward a higher frequency of the rare G allele among patients with atrial fibrillation. No statistically significant association was demonstrated. Given the exploratory nature of the study, the limited sample size, and potential confounding by age, further large-scale studies are required to clarify the role of *PRRX1* genetic variants in atrial fibrillation susceptibility.

## Figures and Tables

**Table 1:** Initial characteristics of the study participants.

Parameter	Patients with AF (n = 75)	Healthy Patients (n = 73)	Patients with AH and CHD Without AF (n = 50)	*p*-Level
Age, years	66.76 ± 9.13	54.32 ± 8.63	64.96 ± 9.74	0.017 *
Male, n (%)	43 (57.3)	35 (47.9)	31 (62.0)	0.6 **
Female, n (%)	32 (42.7)	38 (52.1)	19 (38.0)	0.6 **
Weight, kg	81.0 ± 13.85	73.4 ± 12.27	80.4 ± 13.67	0.866 *
Height, cm	166.60 ± 8.66	165.4 ± 8.26	165.98 ± 9.46	0.668 *
Smoking, n (%)	12 (16.0)	9 (12.3)	6 (12.0)	0.53 **
Alcohol, n (%)	8 (10.7)	6 (8.2)	2 (4.0)	0.18 **
Cholesterol, mmol/L	4.67 ± 1.24	4.66 ± 1.28	4.91 ± 1.23	0.394 *
LDL, mmol/L	2.92 ± 1.04	2.88 ± 0.72	2.94 ± 0.83	0.810 *

Note: n—the number of patients, LDL—low-density lipoproteins, *p* ≥ 0.05, *—Kruskal–Wallis test, **—Pearson chi-square.

**Table 2:** Frequency distribution of genotypes and the A/G alleles of the *rs3903239* polymorphism of the *PRRX1* gene in the main and the control groups.

Genotypes	Main Group (n = 75)		Control Group 1 (n = 73)		Control Group 2 (n = 50)	
	abs.	%	abs.	%	abs.	%
AA	25	33.3	33	45.2	17	34
AG	39	52	31	42.5	24	48
GG	11	14.7	9	12.3	9	18
Total	75	100	73	100	50	100

**Table 3:** Frequency distribution of the A/G alleles of the *rs3903239* polymorphism of the *PRRX1* gene in the main and the control groups.

Alleles	Main Group (n = 75)		Control Group 1 (n = 73)		Control Group 2 (n = 50)	
	abs.	%	abs.	%	abs.	%
A	89	59.3	97	66.4	58	58
G	61	40.7	49	33.6	42	42
Total	150	100	146	100	100	100

**Table 4:** Frequency distribution of the A/G alleles of the *rs3903239* polymorphism of the *PRRX1* gene in the main and the control group 1.

Alleles	Main Group (n = 75)		Control Group 1 (n = 73)		OR 95% CI	
	abs.	%	abs.	%	Lower Limit	Upper Limit
A	89	59.3	97	66.4	0.845	2.178
G	61	40.7	49	33.6	1.357	
Total	150	100	146	100		

## Data Availability

The original contributions presented in this study are included in the article. Further inquiries can be directed to the corresponding author.
